# Catalytic Reduction of Hexavalent Chromium Using Iron/Palladium Bimetallic Nanoparticle-Assembled Filter Paper

**DOI:** 10.3390/nano9081183

**Published:** 2019-08-20

**Authors:** Daniel Shi, Zhijun Ouyang, Yili Zhao, Jie Xiong, Xiangyang Shi

**Affiliations:** 1 Zhejiang Provincial Key Laboratory of Fiber Materials and Manufacturing Technology, College of Materials and Textiles, Zhejiang Sci-Tech University, Hangzhou 310018, China; 2 College of Chemistry, Chemical Engineering and Biotechnology, Donghua University, Shanghai 201620, China

**Keywords:** filter paper, Fe/Pd nanoparticles, Cr(VI), catalysis

## Abstract

Iron/palladium bimetallic nanoparticles (Fe/Pd NPs) are important catalytic materials for the field of environmental remediation. In the present study, filter paper was employed as a substrate for the assembly of Fe/Pd NPs and further applied for the catalytic conversion of hexavalent chromium Cr(VI) toward trivalent Cr(III). First, a filter paper with negative charge was assembled with a layer of positively charged polyethylenimine (PEI) through electrostatic interaction; then, the abundant amine groups of PEI were used to complex Fe(III) ions, followed by reduction via sodium borohydride to produce an Fe NP-assembled filter paper. Thereafter, the Fe/Pd NPs were produced by the reduction of PdCl_4_^2−^ through Fe NPs. The prepared filter paper assembled with Fe/Pd NPs with a mean diameter of 10.1 nm was characterized by various techniques. The Fe/Pd NP-assembled filter paper possesses powerful catalytic activity and can be used to transform Cr(VI) to Cr(III). With its low cost, high sustainability, and convenient industrialization potential, the developed approach may be extended to produce other bimetallic NP-immobilized filter paper for different environmental remediation applications.

## 1. Introduction

Hexavalent chromium Cr(VI) has been classified as class I carcinogen that is highly toxic, mutagenic, and carcinogenic by the International Agency for Research on Cancer [1]. The contamination of Cr(VI) in water is mainly caused by industrial activities such as tanning, electroplating, stainless steel manufacturing, wood preservation, and the production of pigments, paints, paper, and pulp [2]. In order to safeguard human health, the World Health Organization stipulates that the maximum concentration of Cr(VI) in drinking water must not exceed 0.05 mg/L [3]. In contrast to highly toxic, soluble and mobile Cr(VI), chromium ions in trivalent state (Cr(III)) have low toxicity and poor fluidity, and are prone to precipitate or adsorb on various adsorbents. Therefore, the conversion of Cr(VI) to Cr(III) is the key step to improve the total chromium removal rate in wastewater.

Over the past few years, considerable efforts have been dedicated to improve techniques to reduce Cr(VI) contamination [4,5,6]. In particular, iron nanoparticles (NPs) are eco-friendly, efficient, and cost-effective for the remediation of Cr(VI) [7,8,9]. However, iron NPs are prone to be oxidized to ferrous ion and form precipitates such as FeOOH, Fe_3_O_4_, Fe(OH)_2_, Fe(OH)_3_, and FeCO_3_ under the existence of water and/or oxygen, which dramatically decreases their reduction rate over time [10]. The introduction of a second kind of metal such as Pd, Ni, Zn, Cu, or Ag onto the surface of iron NPs helps to reduce Cr(VI) and protect the iron NP surface from corrosion [11]. For instance, the improvement of the catalytic capacity of iron/palladium bimetallic NPs (Fe/Pd NPs) may be because the generated H_2_ from iron corrosion is intercalated into the lattice of the Pd layer, and may be dissociated into a powerful reductant such as H [12].

Despite tremendous evidence indicating the great role of Fe/Pd NPs for environmental remediation, the major concern is that Fe/Pd NPs suffer from the problem of easy agglomeration due to direct interactions between particles, thus resulting in decreased reactivity. Besides, the recovery of colloidal Fe/Pd NPs is difficult after the treatment of contaminated water, which greatly hinders their practical applications in environmental remediation. To solve the aforementioned problems, numerous materials including activated carbon [13], membranes [14,15], resin [12], and nanofibrous mats [16] have been utilized to support Fe/Pd NPs. However, the development of a new and cost-effective strategy to immobilize Fe/Pd NPs to improve the performance of environmental remediation remains a great issue.

Filter paper is an inexpensive, biodegradable, and renewable material that exhibits excellent performance in a large number of applications [17,18,19]. Cellulose is the main raw material of filter paper, which has a wide range of sources, and the oxygen-rich groups endow it with easy functionalization. Besides, the cellulose with a microscale fibrous structure makes filter paper highly porous and mechanically durable. In our previous studies, Au or Ag NPs were synthesized by using branched polyethylenimine (PEI) as a template, and then assembled onto the cellulose microfibril surface [20,21]. We also demonstrated that the negatively charged cellulose microfibril could be firstly modified with PEI through electrostatic adsorption, and then complexed with PdCl_4_^2−^ ions for generating Pd NPs [22]. These earlier studies related to the functionalization of metallic NPs on the surface of filter paper lead us to assume that bimetallic Fe/Pd NPs can also be functionalized onto the surface of cellulose microfibril for environmental remediation.

In the present study, we utilized filter paper as a substrate to support Fe/Pd NPs for the subsequent catalytic conversion of Cr(VI) to Cr(III). Firstly, the surface of filter paper was electrostatically adsorbed with a layer of PEI to endow it with abundant amine groups. Then, the filter paper assembled by PEI was complexed with Fe(III) ions, and reduced by sodium borohydride (NaBH_4_) to form zero-valent Fe NPs. Finally, the filter paper containing Fe NPs was dipped into a solution of PdCl_4_^2−^ ions to produce Fe/Pd NPs through the reduction of PdCl_4_^2−^ ions with Fe NPs. The formed Fe/Pd NP-assembled filter paper was thoroughly characterized. The catalytic activity and reusability of the produced Fe/Pd NP-assembled filter paper were tested by transforming Cr(VI) to Cr(III). Through in-depth literature exploration, we can safely claim that there has been no similar study on the preparation of Fe/Pd NP-assembled filter paper for catalytic applications.

## 2. Experimental Section

### 2.1. Materials

Filter paper, ferric chloride (FeCl_3_), potassium dichromate (K_2_Cr_2_O_7_) and formic acid were obtained from Sinopharm Chemical Reagent Co., Ltd. (Shanghai, China). Branched polyethylenimine (PEI, M_w_ = 25 000) was acquired from Aldrich (St Louis, MO). Sodium borohydride (NaBH_4_) and potassium palladium chloride (K_2_PdCl_4_) were purchased from J&K Chemical Ltd. (Beijing, China). Water used in all experiments was purified using a Milli-Q Plus 185 water purification system (Millipore, Bedford, MA) with a resistivity higher than 18.2 MΩ·cm.

### 2.2. Preparation of Fe/Pd NP-Assembled Filter Paper 

**Scheme 1:**
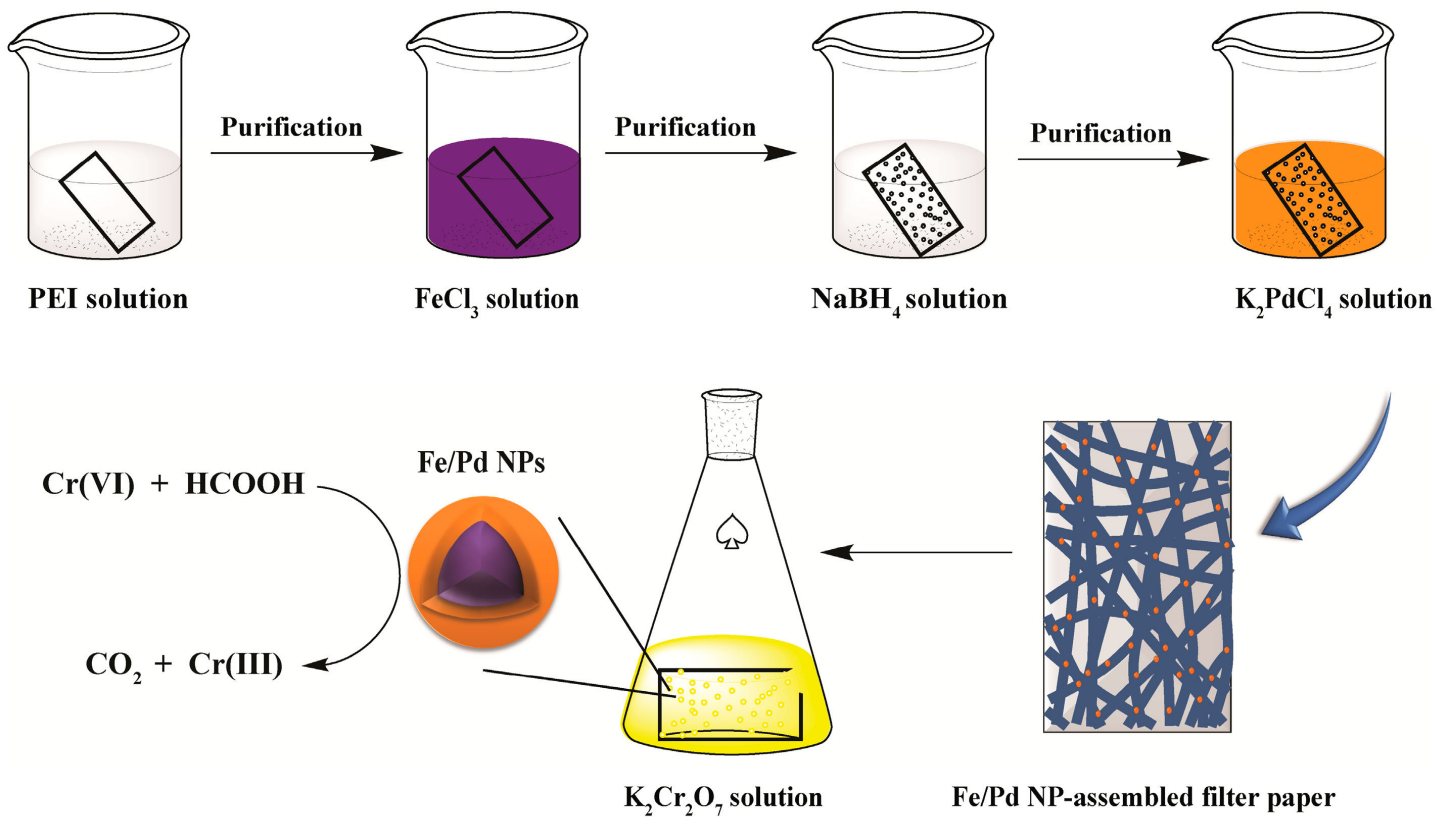
Schematic diagram of the formation of bimetallic iron/palladium nanoparticle (Fe/Pd NP)-assembled filter paper for transforming hexavalent chromium (Cr(VI)) to chromium irons in trivalent state (Cr(III)).

The procedure to prepare Fe/Pd NP-assembled filter paper was illustrated in Scheme 1. First, a piece of rectangular filter paper (1 × 2 cm^2^, 18 mg) was immersed into an aqueous solution of PEI (20 mg/mL, 5 mL) for 30 min, followed by washing three times with water to remove the extra PEI. Then, the PEI-assembled filter paper was immersed in FeCl_3_ aqueous solution (0.4 μM, 5 mL) under continuous shaking for 3 h, and rinsed with water. Thereafter, the Fe(III) ions that were complexed onto PEI-assembled filter paper were reduced by icy cold water solution of NaBH_4_ (0.94 M, 5 mL) for 30 min to form Fe NPs. Finally, the Fe NP-assembled filter paper was reacted with K_2_PdCl_4_ aqueous solution (0.04 μM, 5 mL) for 1 h in order to produce Fe/Pd bimetallic NPs. The prepared Fe/Pd NP-assembled filter paper was thoroughly rinsed with water, vacuum dried, and stored for future use.

### 2.3. Characterization Techniques

The fabricated Fe/Pd NP-assembled filter paper was characterized with various techniques including TM-100 scanning electron microscope (SEM, Hitachi, Tokyo, Japan), JEM2100 transmission electron microscope (TEM, JEOL, Tokyo, Japan), TG 209 F1 thermogravimetric analysis (TGA, NETZSCH Instruments Co., Ltd., Selb/Bavaria, Germany), and Prodigy inductively coupled plasma-optical emission spectroscopy (ICP-OES, Teledyne Leeman Labs, Hudson, NH).

### 2.4. Catalytic Activity Evaluation

The catalytic activity and reusability of the formed Fe/Pd NP-assembled filter paper were assessed through the conversion of Cr(VI) to Cr(III) according to our previous studies [22,23].

## 3. Results and Discussion

### 3.1. Preparation and Characterization of Fe/Pd NP-Assembled Filter Paper

In this study, we utilized filter paper as a substrate to support Fe/Pd NPs. First, filter paper with negative charge was assembled with a layer of PEI via electrostatic interaction; then, Fe(III) ions were complexed with abundant amine groups of PEI, followed by the formation of Fe NPs onto the filter paper after NaBH_4_ reduction. Finally, PdCl_4_^2−^ was reduced by Fe NPs to produce Fe/Pd NPs onto the filter paper. The color of filter paper changes to dark after the assembly of Fe/Pd NPs, indicating the successful formation of Fe/Pd NPs onto filter paper (Figure S1, Supporting Information).

**Figure 1:**
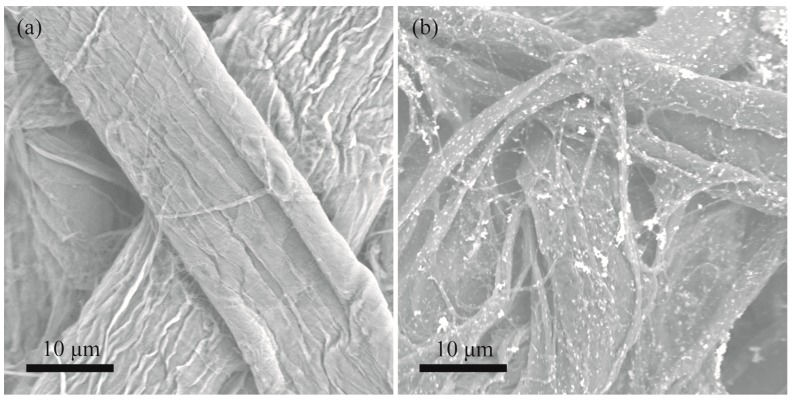
Scanning electron microscopy (SEM) images of the (**a**) iron/palladium (Fe/Pd)-free and (**b**) Fe/Pd nanoparticle (NP)-assembled filter paper.

The morphology of the Fe/Pd NP-assembled filter paper was observed by SEM (Figure 1). It is obvious that the cellulose exhibits a microscale fibrous structure both in Fe/Pd-free and Fe/Pd NP-immobilized filter paper, verifying that the sequential modification procedure does not significantly change the cellulose fibrous structure of the filter paper. Moreover, a great number of white NPs can be clearly seen on the surface of Fe/Pd NP-immobilized cellulose microfibril, confirming the successful formation of the Fe/Pd NPs. Furthermore, it seems that the Fe/Pd NPs distributed homogeneously onto the filter paper surface. In addition, the successful formation of Fe/Pd NPs was also demonstrated by ICP-OES, where the element of Fe and Pd can all be detected. The loading rates of Fe and Pd onto Fe/Pd NP-assembled filter paper were calculated to be 10.11% and 0.25%, respectively. Clearly, the loading rate of Pd is much smaller than that of the single Pd NP-assembled filter paper reported in our previous work [22]. 

**Figure 2:**
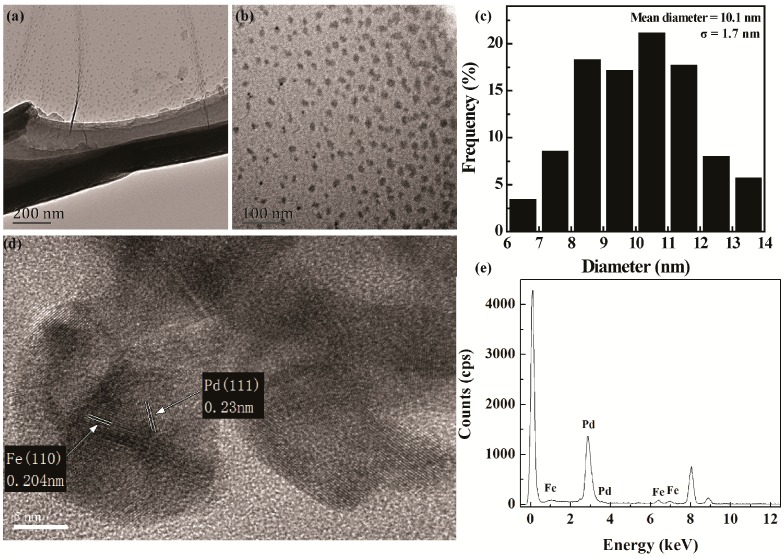
(**a**) Transmission electron microscopy (TEM) image, and (**b**) enlarged TEM image of the iron/palladium nanoparticle (Fe/Pd NP)-assembled filter paper from the cross-section. (**c**) Diameter distribution histogram of Fe/Pd NPs. (**d**) High-resolution TEM image of the Fe/Pd NPs. (**e**) Energy-dispersive spectrum (EDS) spectrum of the Fe/Pd NPs.

TEM was further conducted for the observation of Fe/Pd NPs distribution onto filter paper from the cross-section (Figure 2). It can be seen that individual Fe/Pd NPs with a quasi-spherical shape are densely and uniformly distributed along the cross-section of the filter paper (Figure 2a,b). The mean diameter of the Fe/Pd NPs was measured to be 10.1 ± 1.7 nm (Figure 2c), indicating that the filter paper employed in this study is a good substrate for the growth and assembly of bimetallic Fe/Pd NPs. High-resolution TEM image reveals the lattice structure of the NPs, and the d spacing was calculated to be 0.204 nm and 0.23 nm, corresponding to the [110] plane of α-Fe^0^ and the [111] plane of the face-centered cubic crystal structure of Pd, respectively [24,25]. Furthermore, energy-dispersive spectrum (EDS) shows clearly the signals of Pd and Fe elements, confirming the bimetallic nature of the Fe/Pd NP-assembled filter paper.

**Figure 3:**
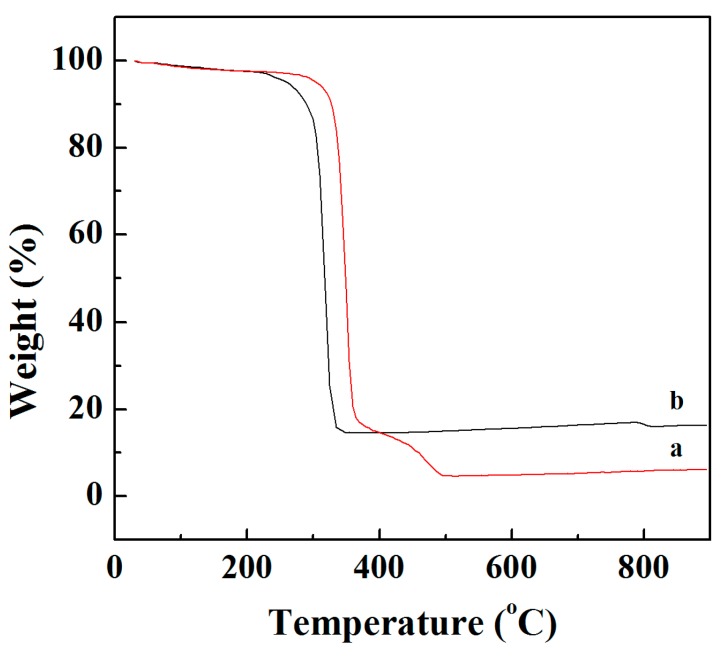
Thermogravimetric analysis (TGA) curves of the (Curve a) iron/palladium (Fe/Pd)-free and (Curve b) Fe/Pd nanoparticle (NP)-assembled filter paper.

The content of Fe/Pd NPs assembled onto filter paper was quantified by TGA (Figure 3). At the beginning, the filter paper with and without Fe/Pd NPs shows slight weight loss as a consequence of water evaporation. With the increasing of temperature, the weight decreased sharply for both the Fe/Pd-free and Fe/Pd NP-assembled filter papers as a result of the decomposition of cellulose and PEI. By comparing the final weight of the Fe/Pd-free filter paper (Figure 3, Curve a) and Fe/Pd NP-assembled filter paper (Figure 3, Curve b), the loading percentage of Fe/Pd NPs was calculated to be 10.12%. Moreover, ICP-OES analysis was also performed to confirm the content of Fe/Pd NPs on the filter paper. It is shown that the loading rate of Fe/Pd NPs is 10.36%, which is quite consistent with the TGA results. 

### 3.2. Catalytic Reduction of Cr(VI)

The potential application of the Fe/Pd NP-assembled filter paper toward the catalytic reduction of Cr(VI) under the existence of formic acid was explored. The transition from Cr(VI) to Cr(III) was confirmed by the gradual color change of the reaction solution. It is obvious that the yellowish color of the reaction solution was fading away as the time increased, and became colorless within 28 min, indicating the effective reduction of Cr(VI) (Figure S2a, Supporting Information). In contrast, the reaction solution treated with the Fe/Pd-free filter paper did not show a distinct color change (Figure S2b, Supporting Information).

**Figure 4:**
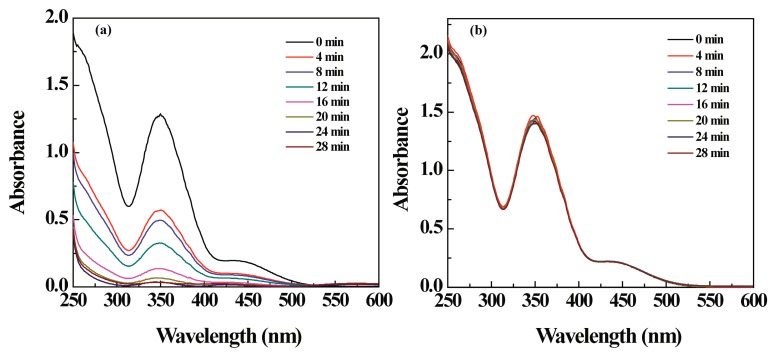
UV-vis spectra of the reaction solution treated with (**a**) iron/palladium nanoparticle (Fe/Pd NP)-assembled filter paper and (**b**) Fe/Pd-free filter paper at different time intervals.

In addition, UV-Vis spectroscopy was applied to monitor the catalytic reduction process (Figure 4). It is clear that the characteristic absorption peak of Cr_2_O_7_^2−^ at 350 nm gradually decreased with the extension of time (Figure 4a), validating the successful reduction of Cr(VI) by the Fe/Pd NP-assembled filter paper. However, for the control group of Fe/Pd-free filter paper, the absorption peak of Cr_2_O_7_^2−^ did not show any significant changes, and the slight decrease of the absorption peak was likely due to the physical absorption of Cr(VI) onto the cellulose microfibril surface. This confirms that in this case, the filter paper does not possess catalytic activity (Figure 4b). Therefore, it can be concluded that the strong catalytic capacity of the filter paper toward the reduction of Cr(VI) is solely resulting from the assembled Fe/Pd NPs. Meanwhile, the designed bimetallic Fe/Pd NPs could significantly decrease the utilization of the expensive Pd element (0.25%) without affecting their catalytic performance, thereby facilitating their industrial applications in the future.

**Figure 5:**
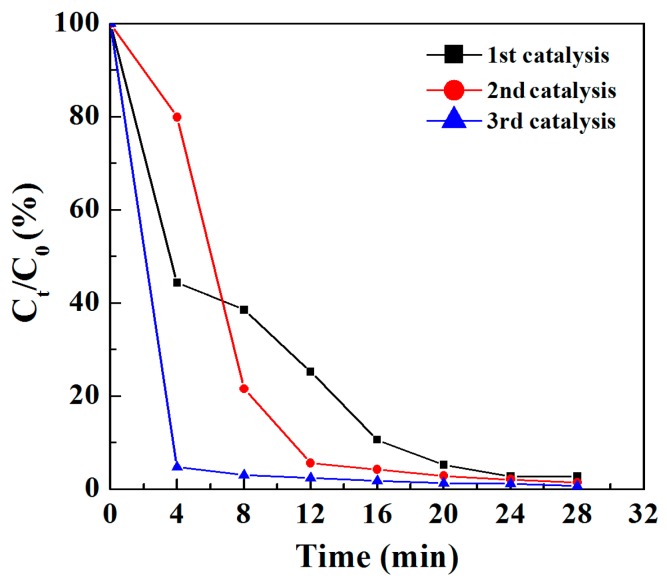
Residual fraction of chromium (VI) (Cr(VI)) versus time during the first, second, and third cycle of catalytic reaction using iron/palladium nanoparticle (Fe/Pd NP)-assembled filter paper.

The reusability of a catalyst must be considered in order to promote its industrial applications. In this present study, the residual fraction of Cr(VI) as a function of reaction time was investigated. After one cycle of catalytic reaction, the Fe/Pd NP-assembled filter paper was washed and dried before the next round of catalytic reaction. It is clear that the residual fraction of Cr(VI) after being catalyzed by Pd/Fe NP-assembled filter paper for 8 min is all less than 40% during the respective three catalytic reaction cycles, whereas for the Pd NP-assembled filter paper, the remaining fractions of Cr(VI) after reacting for 8 min are 35.7%, 45.4%, and 59.5%, respectively for the first, second, and third cycle of catalytic reaction [22]. This indicates that the bimetallic Fe/Pd NP-immobilized filter paper has a better catalysis efficiency than the single Pd NP-assembled filter paper, which is probably due to the synergistic effect of bimetallic Fe/Pd NPs, where Fe core NPs may partially serve as a reducing agent to reduce Cr(VI) to Cr(III). It was calculated that 97.4%, 98.6%, and 99.4% of Cr(VI) could be transformed to Cr(III) within 28 min for the first, second, and third catalytic cycle (Figure 5), demonstrating the outstanding catalytic reusability of the Fe/Pd NP-assembled filter paper. Interestingly, the catalytic efficiency of the bimetallic filter paper slightly increases with the increase of the catalytic cycle number, which may be because more H resulting from H_2_ dissociation exists in the lattice of the Pd layer to support the powerful reduction process. In addition, the stability of the Fe/Pd NP-assembled filter paper was assessed during the repeated catalytic reaction. ICP-OES analysis indicates that no Fe and Pd elements can be detected, even after the third cycle of catalytic reaction, showing that Fe/Pd NPs are stable and are not released from the filter paper during the reaction.

## 4. Conclusions

In conclusion, we successfully utilized filter paper as a substrate to immobilize Fe/Pd bimetallic NPs for transforming Cr(VI) to Cr(III). Branched PEI was firstly electrostatically adsorbed onto the cellulose microfibril of the filter paper, facilitating the following complexation of Fe(III) ions and the formation of Fe NPs. The second metal Pd was further introduced for the formation of Fe/Pd NPs. It is demonstrated that Fe/Pd NPs with a mean diameter of 10.1 nm are homogeneously distributed onto the filter paper surface with a loading percentage of 10.12%. Moreover, the Fe/Pd NP-assembled filter paper could be applied to reduce Cr(VI) with high efficiency and great reusability. With the low cost, degradability, wide availability, porous structure, and durable mechanical property, the filter paper may be extended to support other bimetallic or trimetallic NPs for various environmental remediation applications.

## Supplementary Materials

The following are available online at https://www.mdpi.com/2079-4991/9/8/1183/s1, Figure S1: Photograph of the filter paper without (a) and with (b) the assembly of Fe/Pd NPs, Figure S2: Photograph of the K_2_Cr_2_O_7_ solution treated with (a) Fe/Pd NP free and (b) Fe/Pd NP-assembled filter paper at different time intervals.
