# Supplementary material for: Catalytic Reduction of Hexavalent Chromium Using Iron/Palladium Bimetallic Nanoparticle-Assembled Filter Paper
Source: Nanomaterials (Basel). 2019 Aug 20;9(8):1183. doi: 10.3390/nano9081183 (PMC6724082; doi:10.3390/nano9081183)
Supplement: Supplementary file 1 [file nanomaterials-09-01183-s001.pdf]

nanomaterials

Supplementary Materials

Catalytic Reduction of Hexavalent Chromium Using Rron/Palladium Bimetallic Nanoparticle-Assembled Filter Paper

Daniel Shi [1], Zhijun Ouyang [2], Yili Zhao [1,*], Jie Xiong [1,*], Xiangyang Shi [2,*]

[1] Zhejiang Provincial Key Laboratory of Fiber Materials and Manufacturing Technology, College of Materials and Textiles, Zhejiang Sci-Tech University, Hangzhou, 310018, China

[2] College of Chemistry, Chemical Engineering and Biotechnology, Donghua University, Shanghai 201620, China

* Correspondence: yzhao@zstu.edu.cn (Y.Z.); jxiong@zstu.edu.cn (J.X.); xshi@dhu.edu.cn (X.S.); Tel.: +86-21 67792656 (X.S.)

Received: 24 July 2019; Accepted: 16 August 2019; Published: date

1. Part of Experimental Section

1.1. Materials

Filter paper, ferric chloride ($FeCl_3$), potassium dichromate ($K_2Cr_2O_7$) and formic acid were obtained from Sinopharm Chemical Reagent Co., Ltd. (Shanghai, China). Branched polyethylenimine (PEI, Mw = 25 000) was acquired from Aldrich (St Louis, MO). Sodium borohydride ($NaBH_4$) and potassium palladium chloride (K_2PdCl_4) was purchased from J&K Chemical Ltd. (Beijing, China). Water used in all experiments was purified using a Milli-Q Plus 185 water purification system (Millipore, Bedford, MA) with a resistivity higher than 18.2 MΩ·cm.

1.2. Characterization Techniques

Scanning electron microscopy (SEM, TM-100, Hitachi, Tokyo, Japan) was conducted to characterize the morphology of the filter paper with and without the assembly of Fe/Pd NPs at an operating voltage of 10 kV. Before the measurements, samples were sputter-coated with 10 nm thick gold films. Transmission electron microscopy (TEM, JEM2100, JEOL Ltd., Tokyo, Japan) was applied to observe the cross section of the Fe/Pd NP-assembled filter paper at an operating voltage of 200 kV. Prior to the observation, Fe/Pd NP-assembled filter paper was frozen and cut into ultrathin sections by an ultramicrotome. Mean diameter of the formed Fe/Pd NPs was calculated by measuring the diameter of more than 200 Fe/Pd NPs in the TEM images using image analysis software ImageJ 1.40G (http://rsb.info.nih.gov/ij/download.html). The elemental composition of the samples was analyzed by energy-dispersive spectroscopy (EDS) detector attached to the TEM. Thermal gravimetric analysis (TGA, TG 209 F1, NETZSCH Instruments Co., Ltd., Selb/Bavaria, Germany) was performed at a heating rate of 20 °C/min from room temperature to 900 °C in an air atmosphere in order to analyze the percentage of Fe/Pd NPs assembled onto the filter paper. Furthermore, inductively coupled plasma-optical emission spectroscopy (ICP-OES, Hudson, NH) was utilized to confirm the loading percentage of the Fe/Pd NPs onto the filter paper. Briefly, Fe/Pd NP-assembled filter paper with a weight of 5 mg was immersed in aqua regia (1 mL) for 3 h and the extract solution was diluted before analysis.

1.3. Catalysis Experiments

The catalytic efficiency and reusability of Fe/Pd NP-assembled filter paper towards the reduction of Cr(VI) was evaluated according to our previous studies [1, 2]. Firstly, $K_2Cr_2O_7$ solution (3 mM, 10 mL), formic acid (1.5 mL) and water (15 mL) were mixed under magnetic stirring and kept in a water bath with a temperature of 50 °C. Then the pure filter paper or the Fe/Pd NP-assembled filter paper was immersed into the above mixture solution. At the predetermined time point, 0.5 mL of the mixture solution was taken out and diluted to 1.0 mL before analyzing the absorbance of $Cr_2O_7^{2-}$ in the wavelength range of 250–600 nm using a Lambda 25 UV-vis spectrometer (Perkin Elmer, Boston, MA). After one cycle of catalytic reaction, the Fe/Pd NP-assembled filter paper was washed, dried and reused for the next cycle of catalytic reaction.

The residual fraction of Cr(VI) at the predetermined time point during each cycle of catalytic reaction was calculated through the following equation:

$$\text{Residual fraction of Cr(VI)} = C_t/C_0 \times 100\% \quad (1)$$

where C_0 is the initial absorbance of $Cr_2O_7^{2-}$ at 350 nm and C_t the absorbance of $Cr_2O_7^{2-}$ at time t.

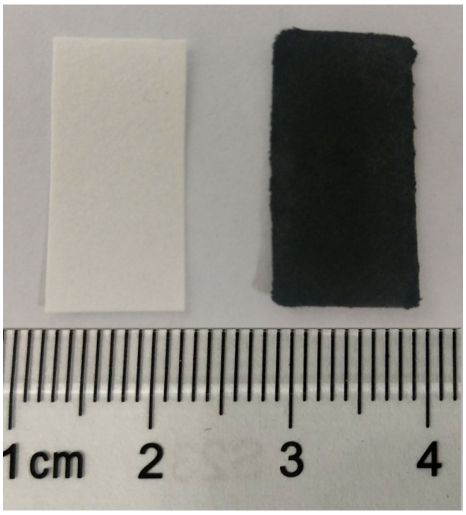

Figure. S1 Photograph of the filter paper without (**a**) and with (**b**) the assembly of Fe/Pd NPs.

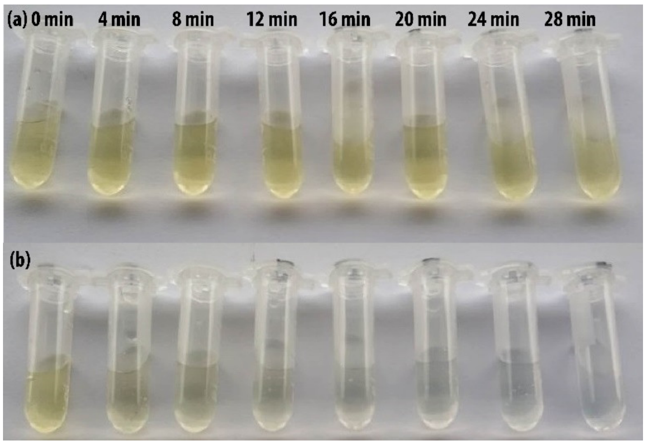

Figure. S2 Photograph of the $K_2Cr_2O_7$ solution treated with (**a**) pure and (**b**) Fe/Pd NP-assembled filter paper at different time intervals.

References

1. Zhao, Y. L.; Liu, L.; Shi, D.; Shi, X. Y.; Shen, M. W. Performing a catalysis reaction on filter paper: development of a metal palladium nanoparticle-based catalyst. *Nanoscale Adv.* **2019**, *1*, 342-346.
2. Huang, Y. P.; Ma, H.; Wang, S. G.; Shen, M. W.; Guo, R.; Cao, X. Y.; Zhu, M. F.; Shi, X. Y. Efficient catalytic reduction of hexavalent chromium using palladium nanoparticle-immobilized electrospun polymer nanofibers. *ACS Appl. Mater. Interfaces* **2012**, *4*, 3054-3061.

© 2019 by the authors. Submitted for possible open access publication under the terms and conditions of the Creative Commons Attribution (CC BY) license (http://creativecommons.org/licenses/by/4.0/).
